# Protein profile of lambs experimentally infected with *Haemonchus contortus* and supplemented with selenium and copper

**DOI:** 10.1186/1756-3305-7-355

**Published:** 2014-08-05

**Authors:** Guilherme Costa Fausto, Felipe Lamberti Pivoto, Márcio Machado Costa, Sônia Terezinha dos Anjos Lopes, Raqueli Teresinha França, Marcelo Beltrão Molento, Antonio Humberto Hamad Minervino, João Batista Teixeira da Rocha, Marta Lizandra do Rêgo Leal

**Affiliations:** Laboratório de Endocrinologia e Metabologia Animal, Departamento de Clínica de Grandes Animais, Hospital Veterinário Universitário, Universidade Federal de Santa Maria (UFSM), Avenida Roraima 1000, CEP 97105-900, Santa Maria, Rio Grande do Sul Brasil; Laboratório de Analises Clínicas Veterinária, Departamento de Clínica de Pequenos Animais, Hospital veterinário Universitário, UFSM, Santa Maria, Brasil; Laboratório de Doenças Parasitárias da Universidade Federal do Paraná, Rua dos Funcionários, 1540, CEP 80035-050, Curitiba, Paraná Brasil; Universidade Federal do Oeste do Pará, (ORCID 0000-0002-6742-3652), Rua Vera Paz, s/n, CEP 68100-000, Santarém, Pará Brasil; Departamento de Ciências da Saúde, Laboratório de Bioquímica Toxicologica, UFSM, Santa Maria, Brasil

## Abstract

**Background:**

Gastrointestinal nematodes cause significant economic losses in the sheep industry, with frequent reports of anthelmintic resistance. Therefore, alternative methods to control these parasites are necessary. Thus, the aim of the present study was to assess the effect of treatment with selenium and copper on the protein profile of sheep that were experimentally infected with *Haemonchus contortus*.

**Methods:**

Twenty-eight lambs were experimentally infected with *H. contortus* and divided into four experimental groups as follow: G1 - untreated animals; G2 - treated with sodium selenite; G3 - treated with copper; G4 - treated with sodium selenite and copper. The serum protein, body weight and egg count per gram of feces (EPG) were assessed at the baseline and after 20, 40, 60 and 80 days. The parasite burden was assessed 80 days after the beginning of the experiment.

**Results:**

Higher levels of total protein and gamma globulin were observed in the lambs treated with sodium selenite and copper on D80. Copper acted as a growth promoter. The copper-supplemented groups exhibited higher daily and total weight gain. The association of selenium and copper altered the protein profile of sheep. Copper and selenium supplementation reduced EPG and worm burden at the end of the experiment. To the best of our knowledge, this is the first study to demonstrate the positive effect of the combined parenteral supplementation of Se and Cu on *H. contortus* infection.

**Conclusions:**

This injectable supplementation could be used as an auxiliary method to control *H. contortus* in sheep.

## Background

Gastrointestinal nematodes are important parasites in sheep flocks [1, 2], which compromise this activity through a marked reduction in weight gain among the animals [3, 4]. Among gastrointestinal nematodes, *Haemonchus contortus* is the most prevalent and important parasite of small ruminants [5, 6]. It is responsible for hematological and biochemical abnormalities such as hypoproteinemia and hypoalbuminemia [7].

There have been reports of anthelmintic resistance in gastrointestinal nematodes worldwide [8, 9] and the search for alternative methods to control these parasites has become extremely important. Protein supplementation induces an increase of globulin and albumin levels in the animals, with a consequent reduction in egg counts per gram of feces and parasite load [10, 11].

One alternative is the use of trace elements, such as selenium, an important element that affects thyroid activity, immune response, defense muscle damage, reproduction, pregnancy and lactation in animals [12]. A number of studies have reported that additional selenium supplementation can increase the globulin and albumin levels in sheep. Copper has also been implicated in increased globular volume levels, reduced egg counts per gram of feces and decreased parasite burden in small ruminants [13].

Copper oxide wire particles have the potential to reduce the establishment and worm fecundity of *Haemonchus contortus*, thereby alleviating the parasite infection by reducing the number of egg-laying nematodes in the abomasum [14, 15]. According to Burke and Miller [16], multiple doses of copper oxide wire particles were as effective as levamisole in controlling *H. contortus*. On the other hand, Waller *et al*., [17] concluded that there was little, if any, benefit in copper supplementation to control parasites among Swedish sheep. Burke and Miller [18] reported that dietary CU sulfate failed to control gastrointestinal nematodes in goats. Furthermore, the studies that reported the effectiveness of copper in controlling nematodes used oral supplementation. Therefore, further studies are required to assess the parenteral administration of copper.

Selenium-deficient diets have reduced resistance to helminth infection [17]. Supplementation with selenium has provided greater antioxidant protection against oxidative stress generated by the experimental infection of lambs with *H. contortus*, as well as providing better antioxidant protection for neutrophils [18, 19].

Information regarding the parenteral administration of Cu or Se in sheep infected with *H. contortus* is limited in the literature. As far as we know, there are no existing studies about the possible synergic action of the two trace elements. Thus, our hypothesis is that injectable mineral supplementation of copper and/or selenium will promote an increase of plasma proteins in sheep and may provide a better defense against parasites. The aim of the present study was to assess the effect of selenium and copper on the protein profile, worm burden and weight gain of lambs that were experimentally infected with *H. contortus*.

## Methods

The use of animals in the present study was approved by the Ethics and Animal Experimentation Committee of the *Universidade Federal de Santa Maria* under protocol number 82/2009.

### Animals and diet

Twenty-eight crossbred Corriedale x Texel five-month old male lambs were used. The animals were kept in holding pens at the Teaching Veterinary Hospital of the *Universidade Federal de Santa Maria* (UFSM). Sheep were purchased from a commercial farm in the city of Santa Maria where they were fed only natural pasture and had a history of *H. contortus* infection. There were no reported deaths caused by this parasite.

The animals were submitted to an adaptation period of 40 days when receiving anthelmintic treatment (two doses at an interval of 15 days) with a combination of Closantel and Albendazole (0.75 mg kg^-1^ and 0.38 mg kg^-1^, respectively; Closalben^®^ Ceva Santé Animale, Paulínia, São Paulo, Brazil). All animals exhibited fecal egg counts of zero after this treatment.

During the adaptation period and throughout the study, sheep were fed at 3.0% body weight (dry matter basis) with a diet consisting of 30% oat hay (*Avena sativa*) and 70% commercial concentrate (Supra Lã 14, Supra^®^, São Leopoldo, Brazil), which were offered three times a day. The diet contained 87.7% dry matter, 11.6% crude protein, 22.2% acid detergent fiber and 1.7% ether extract. The copper and selenium content in the feed given to the animals were determined at the Chemical Analyses Laboratory of the UFSM. However, due to technical issues, the values were below the detection limits.

### Experimental design

The 28 lambs were stratified into four groups of seven animals each and housed in collective pens (one experimental group per pen). After the adaptation period, all of the animals were experimentally infected with third-stage infective larvae of *H. contortus* and the groups were established based on the treatment received: G1 - untreated animals; G2 - animals treated with sodium selenite; G3 - animals treated with copper; G4 - animals treated with sodium selenite and copper.

The protein profile, body weight, eggs per gram of feces (EPG) and worm burden of the animals were assessed for 80 days after the beginning of the experimental infection.

### Experimental infection

*H. contortus* larvae, obtained by the O’Sullivan & Roberts technique [20], were used to infect the animals as described in a previous report [21]. Over a period of 20 days, 500 third-stage infective larvae (L3), in 5 ml of saline, were administered orally to the lambs every second day, totaling 5,000 larvae after 20 days. The first day of infection was designated as day 0 (D0).

### Treatment

The lambs were subjected to intramuscular (IM) treatment with sodium selenite at a dose of 0.2 mg kg^-1^ (Merck Brazil, Jacarepaguá, RJ, Brazil). The copper (copper lactobionate 5.5 mg/ml, copper gluconate 3.1 mg/ml, and copper octadecanoate 0.98 mg/ml) was administered subcutaneously (SC) at a dose of 3.5 mg kg^-1^ (Cuprhormone^®^; Agroinsumos Laboratories, Buenos Aires, Argentina). Sodium selenite supplementation was administered 20 days before D0 and repeated on D0. According to the literature, selenium reaches peak blood level within 60 days of administration [22]. Copper was administered on D0 and 30 days later (D30), since it reaches its sanguine peak earlier [23]. This physiological difference in the metabolism of mineral elements was the reason for the different treatment protocols.

### Collection and sample analysis

Blood samples were collected by jugular venipuncture using Vacutainer^®^ (Becton, Dickinson and Company, San Jose-CA, USA) on day zero, before the experimental infection (D0), and after 20, 40, 60 and 80 days (D20, D40, D60 and D80, respectively). The sera samples were obtained by centrifugation and stored at -70°C for later analysis. The electrophoretic profile of proteins (total protein, albumin, alpha, beta and gamma globulins) was determined by electrophoresis using cellulose acetate strips [24].

On the days of blood collection, the animals were weighed in the morning after fasting for at least 8 hours. Fecal samples were also collected to quantify the eggs per gram of feces (EPG), using the McMaster technique with a sensitivity of 100 EPG [25]. The parasite load was quantified on D80 according to the classical technique [26], which involved the euthanasia of three animals from each group with 1 g of thiopental per animal, followed by the administration of 100 ml of potassium chloride.

### Statistical analysis

The protein profile and body weight data were subjected to analysis of variance (ANOVA), followed by Duncan’s test for multiple comparisons. The EPG values and the worm burden were subjected to logarithmic transformation (log_10_ X +1.5) and later to the student’s t-test, with the significance level set at 5%. The analysis was performed using GraphPad InStat software (GraphPad inc., La Jola, CA, USA).

## Results

Table 1 displays the mean values and the statistical analysis of serum (total protein and albumin). The alpha globulin, beta globulin and gamma globulin results are shown in Table 2. The mean total protein values of G4 were higher (P < 0.05) on D80 when compared with all other groups. The albumin concentration did not exhibit statistical differences between the groups or the time periods.

**Table 1:** **- Mean ± Standard deviation of the serum total protein (TP) and albumin (ALB) in lambs from experimental groups throughout the study**

Groups	D0		D20		D40		D60		D80	
	TP	ALB	TP	ALB	TP	ALB	TP	ALB	TP	ALB
G1	7.98 ± 0.40^Aa^	3.14 ± 0.36^Aa^	7.81 ± 0.49^Aab^	3.30 ± 0.48^Aa^	6.79 ± 0.95^Ab^	3.13 ± 0.47^Aa^	7.30 ± 0.50^Aab^	3.29 ± 0.66^Aa^	6.96 ± 0.92^Bab^	3.12 ± 0.42^Aa^
G2	8.41 ± 0.07^Aa^	3.49 ± 0.32^Aa^	7.36 ± 0.41^ABb^	3.22 ± 0.26^Aa^	7.05 ± 0.80^Ab^	3.20 ± 0.34^Aa^	6.83 ± 0.62^Ab^	3.11 ± 0.35^Aa^	6.99 ± 0.88^Bb^	3.17 ± 0.51^Aa^
G3	7.80 ± 0.47^Aa^	3.24 ± 0.23^Aa^	6.92 ± 0.35^Bb^	3.04 ± 0.28^Aa^	6.66 ± 0.59^Ab^	2.95 ± 0.15^Aa^	6.67 ± 0.49^Ab^	2.98 ± 0.32^Aa^	6.62 ± 0.54^Bb^	3.19 ± 0.40^Aa^
G4	7.64 ± 0.41^Aa^	3.18 ± 0.24^Aa^	7.80 ± 0.58^Aa^	3.42 ± 0.27^Aa^	7.25 ± 0.69^Aa^	3.28 ± 0.37^Aa^	7.45 ± 0.67^Aa^	3.38 ± 0.28^Aa^	7.94 ± 0.76^Aa^	3.43 ± 0.37^Aa^

G1 - infected and untreated animals; G2 - infected and treated with sodium selenite animals; G3 - infected and treated with copper animals; G4 – infected and treated with sodium selenite and copper animals. Different capital letters in the same column indicate a significant difference between the groups (p < 0.05). Different lowercase letters on the same line indicate a significant difference between the experimental periods within a group (p < 0.05).

**Table 2:** **- Mean ± Standard deviation of the serum alpha globulins (α), beta globulins (β) and gamma globulins (μ) in lambs from experimental groups throughout the study**

Experimental periods		G1	G2	G3	G4
D0	α	0.89 ± 0.11^Aa^	0.92 ± 0.15^Aa^	0.83 ± 0.09^Aa^	0.82 ± 0.06^Aa^
	β	0.61 ± 0.12^Aa^	0.59 ± 0.06^Aa^	0.51 ± 0.07^Aa^	0.51 ± 0.06^Aa^
	γ	2.54 ± 0.38^Aa^	2.75 ± 0.35^Aa^	2.58 ± 0.51^Aa^	2.45 ± 0.17^Aa^
D20	α	0.88 ± 0.09^Aa^	0.81 ± 0.12^Aa^	0.77 ± 0.09^Aa^	0.85 ± 0.08^Aa^
	β	0.52 ± 0.08^Aa^	0.49 ± 0.08^Ab^	0.45 ± 0.08^Aab^	0.56 ± 0.13^Aa^
	γ	2.49 ± 0.37^Aa^	2.29 ± 00.21^Ab^	2.20 ± 0.25^Aa^	2.37 ± 0.16^Aa^
D40	α	0.73 ± 0.08^Aa^	0.74 ± 0.09^Aa^	0.73 ± 0.08^Aa^	0.74 ± 0.15^Aa^
	β	0.42 ± 0.90^Aa^	0.42 ± 0.04^Ab^	0.38 ± 0.07^Ab^	0.42 ± 0.06^Aa^
	γ	2.08 ± 0.28^Aa^	2.17 ± 0.31^Ab^	2.10 ± 0.39^Aa^	2.27 ± 0.24^Aa^
D60	α	0.77 ± 0.07^Aa^	0.80 ± 0.12^Aa^	0.71 ± 0.14^Aa^	0.84 ± 0.16^Aa^
	β	0.51 ± 0.17^Aa^	0.47 ± 0.06^Ab^	0.42 ± 0.07^Aab^	0.47 ± 0.10^Aa^
	γ	2.14 ± 0.22^Aa^	1.96 ± 0.26^Ab^	2.04 ± 0.28^Aa^	2.35 ± 0.43^Aa^
D80	α	0.82 ± 0.10^Aa^	0.88 ± 0.89^Aa^	0.83 ± 0.13^Aa^	0.93 ± 0.15^Aa^
	β	0.50 ± 0.16^Aa^	0.47 ± 0.11^Ab^	0.38 ± 0.07^Ab^	0.55 ± 0.14^Aa^
	γ	2.17 ± 0.37^Ba^	2.17 ± 0.25^Bb^	2.15 ± 0.38^Ba^	2.56 ± 0.31^Aa^

G1 - infected and untreated animals; G2 - infected and treated with sodium selenite animals; G3 - infected and treated with copper animals; G4 – infected and treated with sodium selenite and copper animals. Different capital letters on the same line indicate a significant difference between the groups. Different lowercase letters in the same column indicate a significant difference between the experimental periods within a group (p <0.05).

This similarity between the serum concentration in the different groups and experimental periods was also observed for alpha globulin and beta globulin. A significant reduction (p < 0.05) was observed in the levels of beta globulin in lambs from G2 and G3, when compared to D0 of the experiment. The levels of gamma globulin in G2 showed a significant reduction (p < 0.05) on D20, D40, D60 and D80, when compared to D0. On the other hand, gamma globulin levels were significantly higher in G4 (p < 0.05) than in the other groups at the end of the experiment (Table 2).

On D0, sheep from G1, G2, G3 and G4 had an average live weight of 21.50 kg, 22.84 kg, 23.86 kg and 25.35 kg, respectively. This difference was due to stratification based on the previous EPG. At the end of the experiment, the total and daily weight gains were higher (P < 0.05) in the copper supplemented groups (Table 3).

**Table 3:** **- Mean ± Standard deviation of the total and daily weight gain and worm burden of sheep at the end of the experiment (D80)**

Groups	Weight gain (kg)	Daily weight gain (kg)	Worm burden
G1	4.80^b^ ± 1.5	0.060^b^ ± 0.019	568^a^ ± 707,3
G2	5.23^b^ ± 1.5	0.065^b^ ± 0.019	527^a^ ± 421,9
G3	7.49^a^ ± 1.7	0.094^a^ ± 0.021	430^a^ ± 122,9
G4	7.37^a^ ± 0.7	0.092^a^ ± 0.008	365^b^ ± 117,6

G1 - infected and untreated animals; G2 - infected and treated with sodium selenite animals; G3 - infected and treated with copper animals; G4 – infected and treated with sodium selenite and copper animals. Different lowercase letters in the same column indicate a significant difference between the groups (p <0.05).

**Figure 1:**
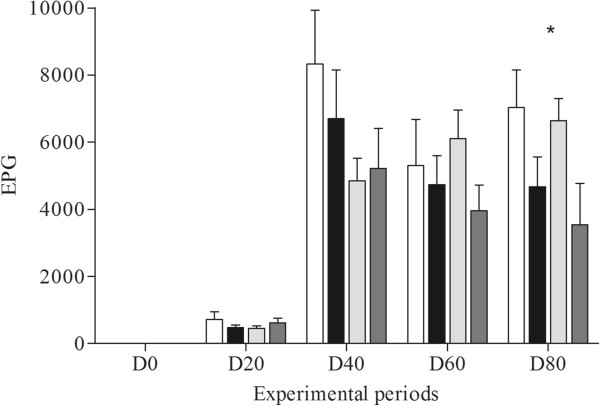
**Egg count per gram of feces (EPG) of lambs experimentally infected with** ***Haemonchus contortus*** **and treated with a source of selenium and copper.** G1 - infected and untreated animals; G2 - infected and treated with sodium selenite animals; G3 - infected and treated with copper animals; G4 – infected and treated with sodium selenite and copper animals. Asterisk in experimental periods indicates a statistical difference (p <0.05) between G4 and the other groups.

On D0, all animals exhibited EPG values equal to zero. These values increased from D20 onwards. At the end of the experiment (D80), lambs from G4 exhibited significantly lower EPG values (p < 0.05), when compared to the other groups (Figure 1). This significant difference was also observed for the mean parasite load, which was 568 in G1, 526 in G2, 430 in G3 and 365 in G4 (Table 3).

## Discussion

*H. contortus* infection in sheep causes disorder in their hematopoietic system, resulting in anemia and severe damage to the abomasal mucosa, including loss of serum proteins [7]. The results of the present study indicated an additive effect of selenium and copper supplementation in lambs from G4, resulting in the reduction of worm burden, which led to less mucosal damage and blood loss, thereby increasing the levels of total protein. This additional action of selenium and copper was clear in the relatively linear relationship of the parasite burden throughout the four groups and by the absence of an increase in serum protein concentration in lambs from G2 and G3, which only received selenium or copper, respectively.

Kumar *et al.*[27] observed no changes in the protein profile of uninfected lambs treated with different concentrations of selenium. Dezfoulian *et al.*[28] found no differences in the red blood cells, hemoglobin and hematocrit of uninfected animals treated with different concentrations of copper. Therefore, the alterations in the protein profile observed in the present study occurred as a result of the parasitic infection and/or the additional effect from both Cu and Se on the immune system in response to the infection.

An increase of serum gamma globulin was observed in lambs from G4, which exhibited significantly higher values (p <0.05) than the other groups on D80, resulting in a reduced EPG (Figure 1) and parasite load (Table 3). These data suggest that supplementation with selenium and copper increased the levels of protein in the blood and reduced parasitic infection among sheep. An efficient immune response is associated with the levels of selenium in the organism [12]. The supplementation of this mineral may improve both the innate and humoral immune response [29]. Other studies of mineral supplementation, such as one that examined the action of zinc on the productive parameters of sheep [30], have reported increased feed efficiency and body weight among lambs, when compared to the control group. The profiles of the other blood proteins analyzed in the present study (albumin, alpha globulin and beta globulin) did not differ significantly between the groups.

Further studies are needed to better understand how Se and Cu affect the fecundity of worms and the ability of the host’s immune system to reduce the number of infecting worms. The authors of the present study believe that Cu and Se supplementation improved the protein profile of sheep and consequently improved both the resistance and resilience of the host against *H. contortus*.

Studies have shown that selenium supplementation provides greater antioxidant protection against the oxidative stress generated by the experimental infection of lambs with *H. contortus*, improving the host immunity [21, 31], and that Se supplementation alters the gene expression profiles associated with innate immunity in whole-blood neutrophils of sheep [32].

Copper is an essential trace element which has an important role in many physiological functions, including humoral or cellular immune responses. A copper deficiency has been associated with impaired macrophage respiratory burst capacity and lower prostaglandin and leukotriene formation, causing a greater risk of infections [33]. Copper supplementation promoted an increase in serum globulin levels in lambs, possibly due to an increase in gamma globulin, which tends to improve the immune response to parasites [28].

Based on the data of the present study, there was an improvement in the weight gain of infected lambs that were treated with copper (G3 and G4) when compared with G1 and G2. This result is similar to that reported in a previous study, in which sheep supplemented with copper exhibited an increased feed conversion rate when compared to untreated groups [28].

All groups exhibited an EPG value of zero on D0, with an increase (p < 0.05) only occurring from D20 onwards (Figure 1). This fact validates the method of experimental infection with *H. contortus* used in the present study, since similar results were obtained in studies that established the infection using different methodologies [34, 35]. After D20, a significant reduction (p < 0.05) was observed in the EPG of the lambs from G4, when compared to the other groups, as well as in the parasite burden recorded at the end of the experiment.

## Conclusions

Selenium and cooper supplementation increased (P < 0.05) the total protein and gamma globulin concentrations and decreased (P < 0.05) the EPG and worm burden. Thus, this injectable supplementation could be used as an auxiliary method to control *H. contortus* in sheep. However, further studies are needed to assess its applicability and economic benefits.
